# Anisotropically biaxial strain in non-polar (112–0) plane In_*x*_Ga_1−*x*_N/GaN layers investigated by X-ray reciprocal space mapping

**DOI:** 10.1038/s41598-017-04854-8

**Published:** 2017-07-03

**Authors:** Guijuan Zhao, Huijie Li, Lianshan Wang, Yulin Meng, Zesheng Ji, Fangzheng Li, Hongyuan Wei, Shaoyan Yang, Zhanguo Wang

**Affiliations:** 1 Key Laboratory of Semiconductor Materials Science, and Beijing Key Laboratory of Low Dimensional Semiconductor Materials and Devices, Institute of Semiconductors, Chinese Academy of Sciences, P.O. Box 912, Beijing, 100083 People’s Republic of China; 2 College of Materials Science and Opto-Electronic Technology, University of Chinese Academy of Sciences, Beijing, 100049 People’s Republic of China

## Abstract

In this study, the indium composition *x* as well as the anisotropically biaxial strain in non-polar *a-plane* In_*x*_Ga_1−*x*_N on GaN is studied by X-ray diffraction (XRD) analysis. In accordance with XRD reciprocal lattice space mapping, with increasing indium composition, the maximum of the In_*x*_Ga_1−*x*_N reciprocal lattice points progressively shifts from a fully compressive strained to a fully relaxed position, then to reversed tensile strained. To fully understand the strain in the ternary alloy layers, it is helpful to grow high-quality device structures using *a-plane* nitrides. As the layer thickness increases, the strain of In_*x*_Ga_1−*x*_N layer releases through surface roughening and the 3D growth-mode.

## Introduction

Nitride-based semiconductors are very promising materials for optoelectronic devices, in particular ultraviolet light emitting diodes (UV-LED)^1, 2^. However, epilayers grown along [0001] *c-axis* of the wurtzite crystal structure suffer from spontaneous and piezoelectric fields which will impair the device performance^3^. Growth along non-polar and semi-polar planes reduces or eliminates these fields and hence increases the radiative recombination efficiency^4, 5^. In the past few years, our group has also been attempting the epitaxial growth of the non-polar (11$\bar{2}$0) GaN, In_*x*_Ga_1−*x*_N and semi-polar (11$\bar{2}$2) GaN^6–10^. To grow high-quality device structures using *a-plane* nitrides, it is essential to fully understand the strain in the ternary alloy layers. However, the *a-plane* In_*x*_Ga_1−*x*_N/GaN heterostructures suffer from anisotropically biaxial in-plane strain which is very complex. Although the isotropically biaxial strain in the c-plane nitride heterostructure has been thoroughly discussed^11, 12^, the discussion on anisotropic biaxial in-plane strain in the *a-plane* heterostructure is very necessary and difficult. The X-ray method offers an essential tool to investigate the structural information of defective and distorted crystals. The X-ray technique is high sensitivity to strain and particularly applicable to evaluate the lattice parameter and strain distribution in the epitaxial film^13^. More importantly, reciprocal space mapping (RSM) by high-resolution X-ray diffraction (HRXRD) is ideally suited to research the detailed structural characterization of imperfect layer crystal structure such as nitrides^12, 14^.

In this paper, the non-polar *a-plane* In_*x*_Ga_1−*x*_N/GaN layers grown on *r-plane* sapphire with different indium content has been thoroughly researched by XRD method. The indium composition profile and strain status in each layers have been evaluated from the RSM. We also illustrate the potential of RSM to extract information on the process of Indium incorporation *x* during In_*x*_Ga_1−*x*_N growth.

## Results and Discussion

Non-polar nitrides encounter anisotropic in-plane strain which results in a distortion of the wurtzite unit cell. Therefore the exact determination of lattice constants and solid phase content *x* in ternary alloys is very difficult. The lattice constants *c* and *a*, ${d}_{(1\bar{1}00)}$, distorted angle *γ*, indium composition *x*, strains (*ε*_*xx*GaN_, *ε*_*yy*GaN_ and *ε*_*zz*GaN_) and stresses (*σ*_*xx*GaN_ and *σ*_*zz*GaN_) of the GaN template layer and *a-plane* In_*x*_Ga_1−*x*_N was estimated following the procedure in refs 10 and 15 are shown in Table 1 and Table 2. The negative and positive signs of the strain and stress indicate compression and tensility, respectively.

**Table 1:** Lattice constants, strain and stress in each direction of GaN template layer grown on *r-plane* sapphire obtained from the XRD results.

coordinate crystallographic axis	*x a*-axis	*y m*-axis	*z c*-axis
Lattice constant (Å)	*d*_1_ = 3.1942	*d*_2_ = 2.7545	*c* = 5.1815
Strain (%)	*ε*_xxGaN_ = +0.1645	*ε*_yyGaN_ = −0.262	*ε*_zzGaN_ = −0.067
Stress (GPa)	*σ*_xxGaN_ = 0	*σ*_yyGaN_ = −0.8686	*σ*_zzGaN_ = −0.3621

**Table 2:** Lattice parameters *a*, *c* and *γ* of (11$\bar{2}$0) In_*x*_Ga_1−*x*_N epilayers and indium composition *x* obtained from XRD.

	*a* (Å)	*c* (Å)	*γ*(deg)	*d*_1_(Å)	*d*_2_(Å)	*x*	ε_xx_(%)	ε_yy_(%)	ε_zz_(%)	σ_yy_ (GPa)	σ_zz_ (GPa)
B	3.2553	5.2776	119.94	3.258	2.818	0.19	+0.0579	−0.064	−0. 136	−0.30757	−0.54112
B1	3.2564	5.2480	119.73	3.27	2.816	0.20	+0.3	−0.258	−0. 805	−1.39458	−3.11393
C	3.3316	5.2906	120.83	3.29	2.897	0.31	−0. 249	+1.441	+1.045	+4.3017	−2.9047
C1	3.3168	5.3685	120.05	3.314	2.873	0.36	−0.018	+0.088	−0.056	+0.2671	−0.14957

Here ${d}_{1}=2{d}_{11\bar{2}0}$ and ${d}_{2}={d}_{1\bar{1}00}$ as showed in Fig. 2.

**Figure 1:**
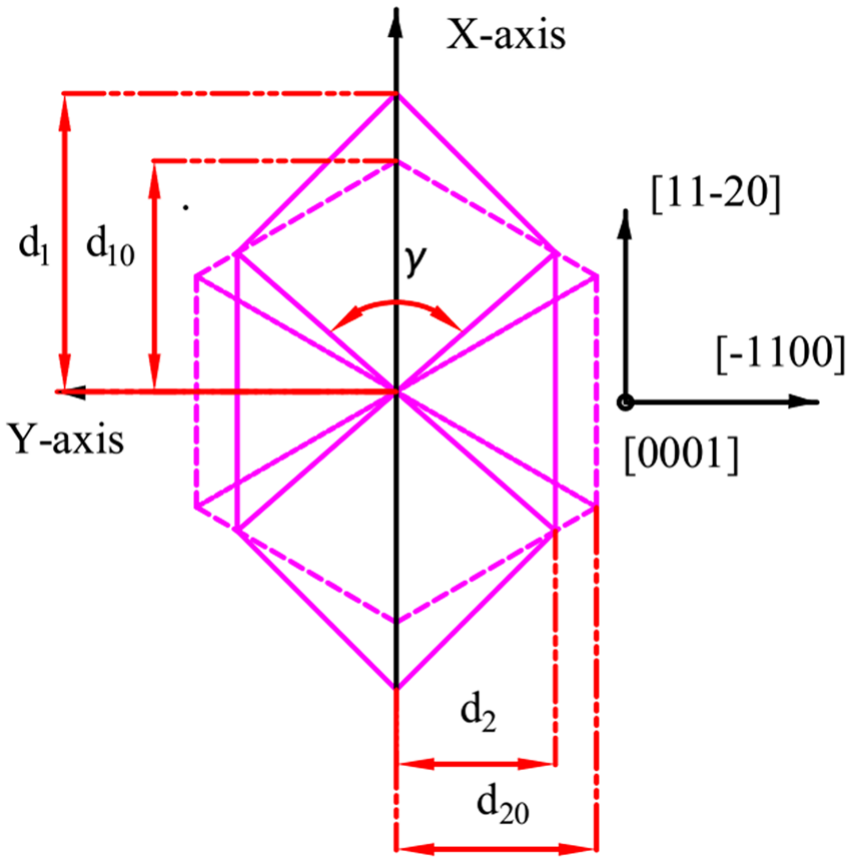
Schematic drawing of distortion of the basal hexagon of the GaN unit cell for GaN film (the undistorted hexagon is shown by dashed line), Here ${d}_{1}=2{d}_{11\bar{2}0}$, ${d}_{2}={d}_{1\bar{1}00}$ and *γ* is the distorted angle.

It is clearly observed that the lattice of the *c-* and *m-axis* directions (in-plane) of the underlying GaN template layer is contracted based on the data in Table 1 (as shown schematically in Fig. 1). It’s worth noting that the stress in the *m-axis* direction is much larger than that in the *c-axis* direction. This is probably caused by the lattice-mismatch between *r-plane* sapphire substrate and *a-plane* (11$\bar{2}$0) GaN epilayer, which are only about 1% along the *c-axis* but 16% along the *m-axis* ^16^.

**Figure 2:**
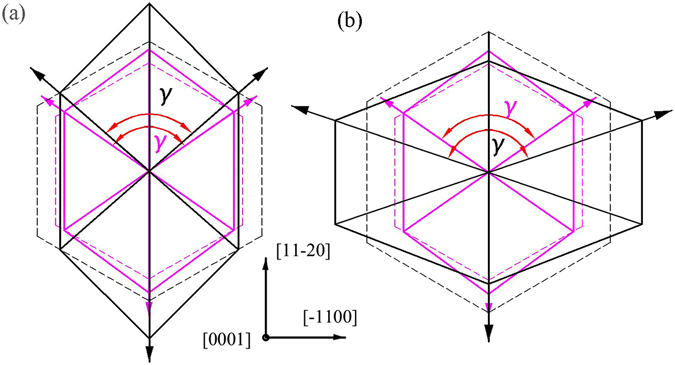
The pink lines and black lines show the basal plane of GaN template and In_x_Ga_1−x_N epilayer unit cell, while the dashed lines and solid lines show the basal plane of perfect and distorted hexagonal unit cell, respectively. (**a**) γ < 120° when indium composition *x* is low, (**b**) γ > 120° when indium composition *x* get higher.

From the estimated results of *a-plane* In_*x*_Ga_1−*x*_N in Table 2, the distorted angle *γ* increases from <120° to >120° with the indium incorporation *x* varies from 0.19 to 0.36. When indium composition *x* is low, the calculation values in the growth direction (*a-axis*) of the In_*x*_Ga_1−*x*_N show a tensile strain as well as these in the two in-plane directions (*m-axis* and *c-axis*) show a compressive strain. The basal hexagon of In_*x*_Ga_1−*x*_N unit cell is distorted as shown schematically in Fig. 2(a). However, when the indium content gets higher, the strain status reverse occurs, i.e. compressive strain in the growth a-direction and tensile strain in the two in-plane directions. The distorted basal hexagon of In_*x*_Ga_1−*x*_N unit cell showed schematically in Fig. 2(b) as well.

**Figure 3:**
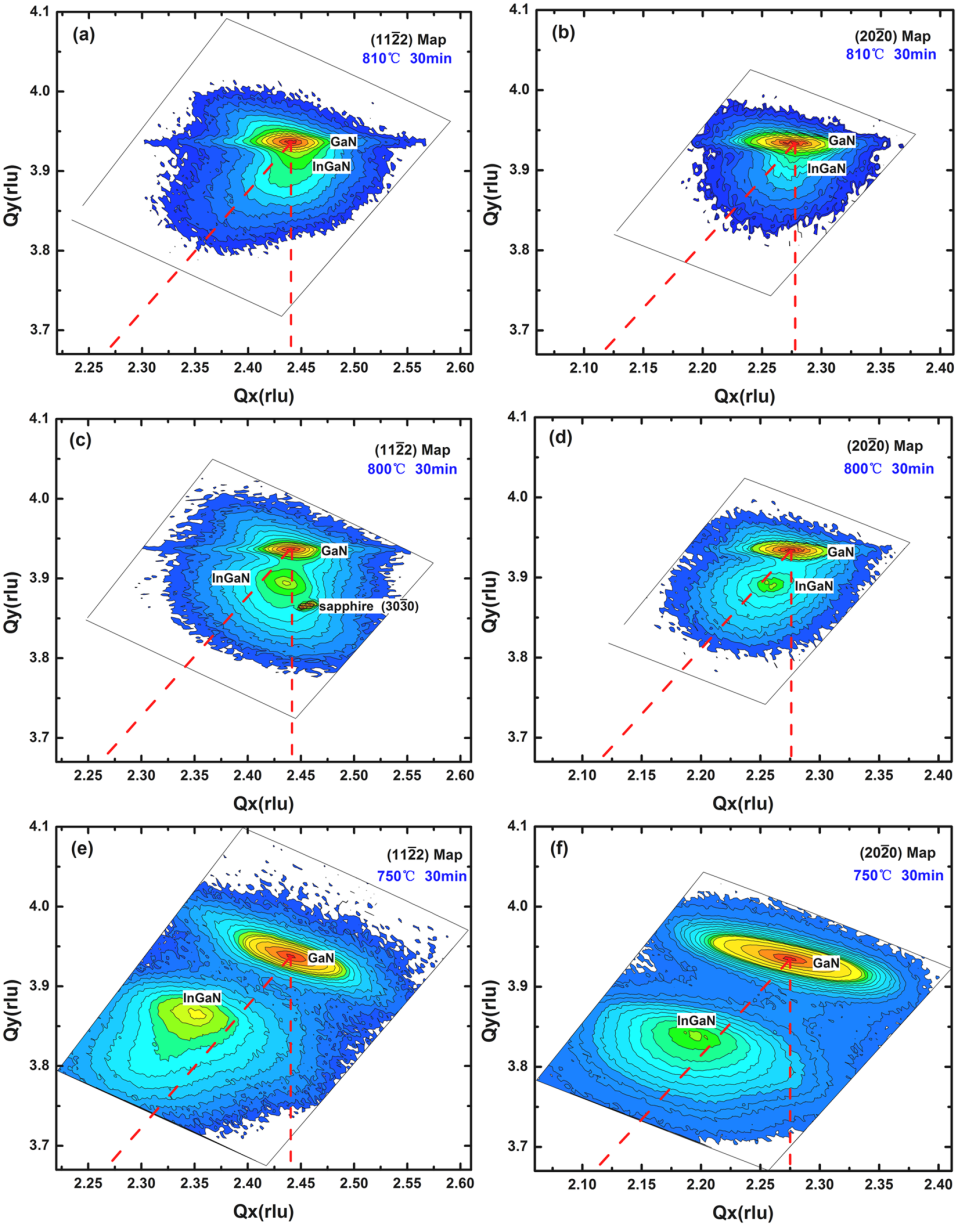
XRD reciprocal lattice space map of *a-plane* In_x_Ga_1−x_N/GaN heterostructure grown on *r-plane* sapphire. (**a**) (11$\bar{2}$2) map of sample A, (**b**) (20$\bar{2}$0) map of sample A, (**c**) (11$\bar{2}$2) map of sample B, (**d**) (20$\bar{2}$0) map of sample B, (**e**) (11$\bar{2}$2) map of sample C, (**f**) (20$\bar{2}$0) map of sample C.

In order to further study the strains in the *a-plane* In_*x*_Ga_1−*x*_N layer, the (11$\bar{2}$2) and (20$\bar{2}$0) asymmetric XRD RSM with high incidence angle configurations have been measured. Figure 3(a–f) shows maps for sample A, B and C, respectively. The abscissas of the (11$\bar{2}$2) and (20$\bar{2}$0) maps are the inverse proportion of the lattice constants *c* and ${d}_{(1\bar{1}00)}$, respectively. The ordinates of both maps are the inverse proportion of the lattice constant *a* ^17^. Two discrete peaks on maps of In_*x*_Ga_1−*x*_N/GaN films are clearly observed, the top peak is determined as a diffraction of GaN template and the lower one is In_*x*_Ga_1−*x*_N alloy. The distance of these two peaks increases with the increasing of Indium composition *x*. Two red dashed lines are drawn from the GaN reciprocal lattice point (RLP): The lines to origin represent the relaxation lines, whereas the perpendicular lines indicate coherent strain on GaN.

For the In_*x*_Ga_1−*x*_N layer at 810 °C (sample A), the diffraction peak of In_*x*_Ga_1−*x*_N is aligned with that of GaN template along the same Q_x_ for both (11$\bar{2}$2) map in Fig. 3(a) and (20$\bar{2}$0) map in Fig. 3(b), which demonstrates that In_*x*_Ga_1−*x*_N is coherently strained to the GaN template. In other words, In_*x*_Ga_1−*x*_N layer is almost fully strained and has no relaxation in both c- and m-direction. The *c-axis* and *m-axis* of the In_*x*_Ga_1−*x*_N film are compressively strained, whereas the *a-axis* of sample A is tensile strained.

In the (11$\bar{2}$2) map for sample B at 800 °C in Fig. 3(c), the diffraction spot of the In_*x*_Ga_1−*x*_N layer is not located exactly above that of the GaN layer. The lattice constant *c* of the In_*x*_Ga_1−*x*_N layer is slightly different from that of GaN. The In_*x*_Ga_1−*x*_N layer was relaxed partially along the *c-axis*. On the other hand, as shown in the (20$\bar{2}$0) map in Fig. 3(d), ${d}_{(1\bar{1}00)}$ of the In_*x*_Ga_1−*x*_N layer is largely different from that of GaN. The stress and strain in the *m-axis* direction is smaller than that in the *c-axis* direction, which is consistent with the calculation results in Table 2. The In_*x*_Ga_1−*x*_N RLPs of sample B are between the fully relaxed and fully strained line, indicated that the *c-axis* and *m-axis* of the In_*x*_Ga_1−*x*_N film are partially compressively strained, whereas the *a-axis* of sample B is tensile strained.

In Fig. 3(e) of the (11$\bar{2}$2) map for sample C (at 750 °C), the diffraction spot of the In_*x*_Ga_1−*x*_N layer is above the fully relaxed line. The lattice constant *c* of the In_*x*_Ga_1−*x*_N layer is larger than the fully relaxed layer, indicating that the In_*x*_Ga_1−*x*_N layer showed a tensile strain along the *c-axis*. On the other hand, as shown in the (20$\bar{2}$0) map in Fig. 3(f), ${d}_{(1\bar{1}00)}$ of the In_*x*_Ga_1−*x*_N layer is also above the fully relaxed line. The In_*x*_Ga_1−*x*_N layer was tensile strained along the *m-axis*. The status of the stress and strain of sample C is different from the sample A and B, which is consistent with the calculation results in Table 2.

As show above, the residual strains in these In_*x*_Ga_1−*x*_N films vary remarkably. The low-In-composition sample A is nearly fully strained, which is compressive strained along the *c-axis* and *m-axis*. For the mid-In-composition sample B (*x* = 0.19), it is partially relaxed. The *c-axis* and *m-axis* of the In_*x*_Ga_1−*x*_N film are partially compressively strained. With the increasing In composition *x*, the In_*x*_Ga_1−*x*_N films are more relaxed because of a larger lattice mismatch with the underlying GaN layer. For the high-In-composition of sample C (*x* = 0.31), the status of the stress and strain are reversed. The *c-axis* and *m-axis* of the In_*x*_Ga_1−*x*_N film are tensile strained. With the increasing of indium composition, the maximum of the In_*x*_Ga_1−*x*_N RLPs progressively shifts from a fully compressive strain position to a fully relaxed position and then to reversed strain position, i.e. tensile strain. It was found that by reducing the growth temperature, the epitaxial layer changes from a pseudomorphic In_*x*_Ga_1−*x*_N with a low indium mole fraction to a relaxed In_*x*_Ga_1−*x*_N with a high indium mole fraction^18^. However, the most interesting result in our study is that when the indium incorporation gets higher, the status of the stress and strain are reversed, i.e. compressive strain in the growth direction and tensile strain in the two in-plane directions.

**Figure 4:**
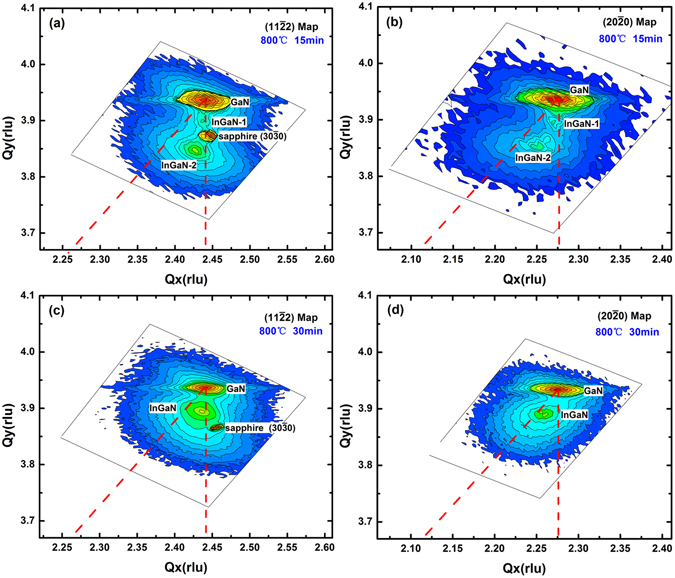
XRD reciprocal lattice space map of *a-plane* In_x_Ga_1−x_N/GaN heterostructure grown on *r-plane* sapphire. (**a**) (11$\bar{2}$2) map of sample B1, (**b**) (20$\bar{2}$0) map of sample B1, (**c**) (11$\bar{2}$2) map of sample B, (**d**) (20$\bar{2}$0) map of sample B.

The (11$\bar{2}$2) and (20$\bar{2}$0) XRD reciprocal lattice space maps of the *a-plane* In_*x*_Ga_1−*x*_N/GaN heterostructures with different growth time of the top In_*x*_Ga_1−*x*_N layer grown at 800 °C are shown in Fig. 4. Figure 4(a,b) show results for the sample B1 (15 min) and Fig. 4(c,d) show results for the sample B (30 min). As shown in Fig. 4(a,b), the lower indium mole fraction InGaN-1 is coherently strained on GaN, whereas the higher indium mole fraction InGaN-2 is partially relaxed. Leyer *et al*. reported that both layers should possess the same strain state in the binodal decomposition (phase separation) case^19^. As the XRD RSMs in Fig. 4 suggest different relaxations state, the multi-peak pattern cannot be explained with phase separation. Therefore, we speculate the multi-peak phenomenon is a result of the strain relaxation process, which is also observed by other groups^19–22^. As the growth proceeds, the InGaN-1 layer with *x*_In_ ~ 0.09 is initially coherently grown on the smooth GaN template, similar to the case of the layer in Fig. 3(a,b). After reaching the critical thickness (about 100 nm)^19^, it should release its accumulated strain through surface roughening/undulation^18, 19^ and/or V-pits^21^ and 3D growth mechanism^22^. The partially relaxed InGaN-2 layer deposites subsequently and as the strain is released by the generation of misfit dislocation or change to 3D growth-mode, the indium mole fraction increases to *x*_In_ ~ 0.20. At the longer growth time of sample B in Fig. 4(c,d), strain relaxation may occur possibly due to the change of growth mode and surface roughness, leading to a single-phase, partially relaxed In_*x*_Ga_1−*x*_N film with high indium mole fraction.

**Figure 5:**
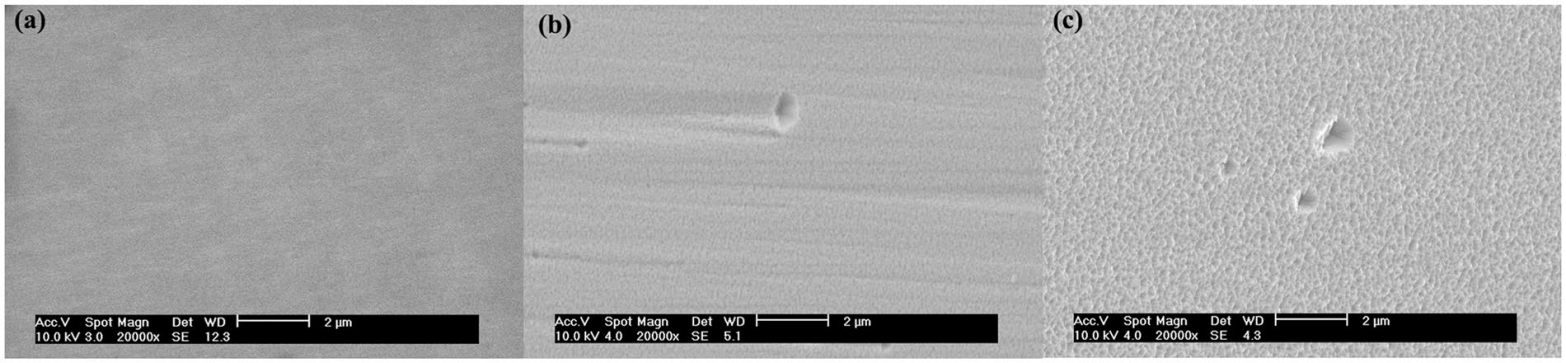
Plan-view SEM images of the *a-plane* GaN template (**a**) and the In_x_Ga_1−x_N/GaN heterostructure ((**b**) sample B1, (**c**) sample B) grown on *r-plane* sapphire.

Figure 5 shows the surface morphologies of the non-polar *a-plane* underlying GaN template layer and In_x_Ga_1−x_N/GaN heterostructures of different growth time so as to understand the correlation between the strain relaxation process and the surface morphology. The surface morphology of the non-polar *a-plane* underlying GaN template (shown in Fig. 2(a)) shows a smooth appearance without any undulating structures. As shown in Fig. 5(b), the surface morphology of sample B1(15 min) exhibits a slight rough surface with many arrowhead-like structures and V-pits. When the thickness of In_x_Ga_1−x_N layer increased, the surface of sample B (30 min) (shown in Fig. 5(c)) shows a very rough surface with high density of V-pits. When a critical thickness/strain is reached, the growth-mode of In_x_Ga_1−x_N transfers from the two dimensional (2D) to the three-dimensional (3D). The accumulated strain of In_x_Ga_1−x_N releases through surface roughening and the 3D islands as the growth time increases.

## Conclusions

In summary, the X-ray diffraction analysis especially the reciprocal space mapping has been shown to be an appropriate tool for the investigation of the strain status and indium composition *x* in non-polar *a-plane* In_*x*_Ga_1−*x*_N/GaN heterostructure. In accordance with XRD RSMs, with increasing indium composition, the maximum of the In_*x*_Ga_1−*x*_N RLPs progressively shifts from a fully compressive strained to a fully relaxed position then to tensile strained. The accumulated strain of In_*x*_Ga_1−*x*_N layers releases through surface roughening and the 3D islands as the layer thickness increases.

## Methods

Non-polar (11$\bar{2}$0) In_*x*_Ga_1−*x*_N/GaN epitaxial layers with different indium compositions *x* were grown on (1$\bar{1}$02) sapphire substrates, using a single 2-inch wafer home-made low-pressure metalorganic chemical vapor deposition (MOCVD) system. The growth was initiated by 1-μm-thick underlying GaN template layer, which use a 40-nm-thick In_*x*_Ga_1−*x*_N interlayer to improve the crystallinity, as discussed in detail elsewhere^6–8^. Subsequently, In_x_Ga_1−x_N alloys with different Indium content *x* were grown at 50 torr by changing the deposition temperature and growth time with Trimethylgallium (TMGa), Trimethylindium (TMIn) and ammonia (NH_3_) as sources and nitrogen as carrier gas. The NH_3_ flow is 3 SLM for all samples at fixed gas phase composition *x*_gas_ [TMIn/(TMGa + TMIn)] = 0.50. There was no cap layer after the growth of In_x_Ga_1−x_N layer and the epilayers were cooled down in NH_3_ ambient. The difference in the growth parameters and In_x_Ga_1−x_N layer thicknesses for samples A–C1 is summarized in Table 3.

**Table 3:** Growth parameters and layer thicknesses of non-polar (11$\bar{2}$0) In_*x*_Ga_1−*x*_N/GaN samples A–C1.

Sample No.	Temperature (°C)	Growth time (min)	Pressure (torr)	TMGa flux (μmol/min)	TMIn flux (μmol/min)	InGaN layer thicknesses (nm)
A	810	30	50	10	10	236
B	800	30	50	10	10	247
B1	800	15	50	10	10	122
C	750	30	50	10	10	292
C1	750	15	50	10	10	180

The structural properties of non-polar *a-plane* In_*x*_Ga_1−*x*_N /GaN samples were examined by HRXRD (Diffuse X-ray Scattering Station of Beijing Synchrotron Radiation Facility), a Huber five-circle diffractometer was used. The radiation energy of the X-ray beam was 8.05 keV with 1.5493 Å of X-ray wavelength and 0.7 × 0.4 mm^2^ (H × V) of the spot size. Field emission scanning electron microscopy (FE-SEM: Sirion) was used to observe the surface morphologies of *a-plane* In_x_Ga_1−x_N/GaN samples.
